# A ubiquitous amino acid source for prokaryotic and eukaryotic cell-free transcription-translation systems

**DOI:** 10.3389/fbioe.2022.992708

**Published:** 2022-09-16

**Authors:** Lakshmeesha K. Nagappa, Wakana Sato, Farzana Alam, Kameshwari Chengan, Christopher M. Smales, Tobias Von Der Haar, Karen M. Polizzi, Katarzyna P. Adamala, Simon J. Moore

**Affiliations:** ^1^ School of Biosciences, University of Kent, Canterbury, United Kingdom; ^2^ Department of Genetics, Cell Biology and Development, University of Minnesota, Minneapolis, MN, United States; ^3^ Centre for Synthetic Biology, Imperial College London, London, United Kingdom; ^4^ Department of Chemical Engineering, Imperial College London, London, United Kingdom

**Keywords:** cell-free gene expression, cell-free protein synthesis, TX-TL, protein production, pichia pastoris cell-free, industrial biotechnology

## Abstract

Cell-free gene expression (CFE) systems are an attractive tool for engineering within synthetic biology and for industrial production of high-value recombinant proteins. CFE reactions require a cell extract, energy system, amino acids, and DNA, to catalyse mRNA transcription and protein synthesis. To provide an amino acid source, CFE systems typically use a commercial standard, which is often proprietary. Herein we show that a range of common microbiology rich media (i.e., tryptone, peptone, yeast extract and casamino acids) unexpectedly provide an effective and low-cost amino acid source. We show that this approach is generalisable, by comparing batch variability and protein production in the following range of CFE systems: *Escherichia coli* (Rosetta^™^ 2 (DE3), BL21(DE3)), *Streptomyces venezuelae* and *Pichia pastoris*. In all CFE systems, we show equivalent or increased protein synthesis capacity upon replacement of the commercial amino acid source. In conclusion, we suggest rich microbiology media provides a new amino acid source for CFE systems with potential broad use in synthetic biology and industrial biotechnology applications.

## Introduction

There is a rising interest in cell-free gene expression (CFE) systems as an enabling technology for prototyping applications and bottom-up approaches to synthetic biology (Sun et al., 2014; Moore et al., 2017b; Garenne et al., 2021). CFE reactions require DNA, crude cell extract, an amino acid (AA) source, and an energy solution, to catalyse mRNA transcription and translation. Interest and potential in CFE systems for industrial biotechnology is growing, with current yields and rates of protein synthesis approaching economic viability for industrial scale-up (Zawada et al., 2011). For recombinant protein production, CFE systems offer a distinct advantage for the synthesis of specialty proteins, peptides or small molecules that are difficult to make in a cell (Zimmerman et al., 2014; Dudley et al., 2020; Meyer et al., 2021; Tian et al., 2022). For example, non-canonical AAs or post-translation modifications (Cui et al., 2020; Charna et al., 2022).

*Escherichia coli* is the dominant and most productive CFE system (Garenne et al., 2021). In addition, several alternative prokaryotic and eukaryotic CFE systems platforms are emerging for distinct applications. The composition of the energy solution, AAs, and other additives is essential for optimising all CFE systems. The energy solution is a complex mixture comprising a primary (e.g., ATP/GTP/CTP/UTP nucleotides) and a secondary energy source, which is typically a high-energy glycolytic pathway intermediate. In addition, cofactors, AAs, macromolecular crowding agents and a range of additives also support the reaction (Gregorio et al., 2019). The primary energy source provides the initial nucleotides and energy to drive mRNA transcription and translation, respectively. In complement, native catabolic enzymes regenerate ATP equivalents from the secondary energy source, which prolongs the CFE time course–up to several hours in batch reactions (Caschera and Noireaux, 2015a; Garenne et al., 2021). If this regeneration cycle is inefficient or absent, some CFE systems use synthetic ATP regeneration systems–e.g., creatine kinase (Anderson et al., 2015). Other limiting factors for CFE systems include non-specific ATP phosphatases, which contribute to energy loss (Calhoun and Swartz, 2007), while other metabolic products (e.g., lactate) can alter the pH and/or inhibit individual enzyme activity (Caschera and Noireaux, 2015a). Therefore, to enhance the reaction length and productivity of CFE systems, the optimisation of the energy solution and energy/product recycling, has been a key focus in recent CFE studies (Shin and Noireaux, 2010; Caschera and Noireaux, 2015a; Anderson et al., 2015; Karim et al., 2018; Garenne et al., 2021). For example, maltodextrin (and maltose) provides a slow-release mechanism to generate glucose, and recycle inhibitory free inorganic phosphate (Wang and Zhang, 2009; Caschera and Noireaux, 2015a). Indeed, several CFE studies highlight the need to optimise L-glutamate (potassium or magnesium salt) as a key variable for achieving maximal CFE (Sun et al., 2013; Cai et al., 2015; Wiegand et al., 2019; Garenne et al., 2021; Moore et al., 2021). This is because L-glutamate is the main nitrogen donor in cells for AA biogenesis, and therefore is the most abundant metabolite in *E. coli* cells, close to 100 mM in concentration (Bennett et al., 2008). In addition, L-glutamate is converted into α-ketoglutarate by L-glutamate transaminase, which leads to ATP production through the Krebs cycle. Therefore, potassium glutamate can provide a sole energy source for industrial scale *E. coli* CFE systems (Zawada et al., 2011). By optimising the various components that contribute to energy recycling, the productivity of CFE systems is enhanced. So far, the maximum *E. coli* CFE batch yields of recombinant protein is 4 mg/ml for the model green fluorescence protein (GFP), using a combination of secondary energy sources including maltodextrin, L-glutamate and d-ribose (Garenne et al., 2021). To increase yields further, metabolite replenishment and removal of waste products via artificial cells increases *E. coli* CFE recombinant protein yields up to 8 mg/ml (Garenne et al., 2021). In summary, energy regeneration and overall metabolism is an important but understudied area of CFE systems.

AAs are a key component of the energy solution, essential for polypeptide synthesis, via tRNA aminoacylation. To provide the 20 canonical AAs, CFE systems rely on expensive commercial AA kits, available as solid or liquid form. While CFE systems can operate without the addition of AAs, this is a limiting factor (Zawada et al., 2011; Moore et al., 2021). For the preparation of AAs there are also some key limitations. First, AAs cost approximately 8% for *E. coli* CFE batch reactions (Sun et al., 2013). Second, most commercial AA solutions are proprietary and contain unknown additives to help solubilise the AAs, which is problematic for some CFE systems (Moore et al., 2018). Third, the manual preparation of single AA stocks from solid powder is time-consuming. Fourth, many AAs have low aqueous solubility (e.g., L-cysteine, L-leucine). To overcome these limitations, Caschera *et al* previously developed a method to solubilise all 20 AAs in concentrated potassium hydroxide (Caschera and Noireaux, 2015b). This method provides an advantage to customise the reaction, by varying the pH and individual AA composition (Caschera and Noireaux, 2015b).

Herein, we show generalisable and yet untapped AA sources to replace commercial AA mixtures in CFE systems. Specifically, we find tryptone, yeast extract, casamino acids and peptone provide effective AA sources for a wide range of CFE systems (e.g., prokaryotic, and eukaryotic). Rich media contain mixtures of AAs, peptides, proteolytic fragments, and sometimes metabolites (e.g., vitamins, primary metabolites), although there is remarkable variability between batches because of the diverse range of sources and extraction methods. In summary, we tested a selection of CFE systems (*E. coli*, *Streptomyces venezuelae* and *Pichia pastoris*) across three cell-free synthetic biology labs (in the United Kingdom and USA), to show the ease of use for rich media as an amino source for general use in CFE systems. We demonstrate specific advantages of this method and identify key research areas that our findings enhance.

## Methods

### Strains, cell-lines, and plasmids

*S. venezuelae* ATCC 10712, *E. coli* Rosetta^™^ 2 (DE3) (Millipore Sigma, 71400-3), and BL21 (DE3) pLysS, *P. pastoris* (also known as *Komagataella phaffi*) X33 overexpressing the *FHL1* gene (Aw and Polizzi, 2019) were used to prepare cell extracts.

### Plasmids

pTU1A-SP44-*mScarlet-I* (AddGene - #163756) plasmid was used at a final concentration of 20 nM in *S. venezuelae* and 10 nM in *E. coli* BL21 (DE3) pLysS systems. pCI-T7Max-UTR1-*deGFP*-8xHis-T500 (AddGene–#178422) plasmid was used for *E. coli* Rosetta^™^ 2 (DE3) CFE reactions, at a final concentration of 10 nM.

### CFE reactions

Original and unmodified CFE protocols were followed for *E. coli* (Sun et al., 2013; Sato et al., 2022), *S. venezuelae* (Moore et al., 2021; Toh et al., 2021) and *P. pastoris* (Spice et al., 2020; Spice et al., 2022). Luminescence measurements were performed as previously described (Aw et al., 2020). Commercial AAs (RTS Sampler kit–Biotech Rabbit, Germany) were used following the manufacturer’s instructions, or individual standards were used as stated within the figure legends. For *E. coli* BL21 (DE3) CFE, the AAs were purchased from Sigma, United Kingdom (LAA21) and prepared as previously described (Moore et al., 2018). For *E. coli* Rosetta^™^ 2 (DE3) CFE, a 20 mM AA stock solution was prepared for the 20 AAs by dissolving in 400 mM potassium hydroxide solution pH 6.5. AAs prepared by this potassium hydroxide method were purchased from MP Biochemicals, with the exception that L-alanine and L-glycine were obtained from Santa Cruz Biotechnology, L-threonine from Sigma-Aldrich and L-arginine from Gold Biotechnology. All cell-free experiments were performed on two independent days to ensure reproducibility, where data is presented as a mean and standard deviation of three technical measurements. Data analysis was performed by GraphPad Prism 9.

### Preparation of the microbiology rich media components for CFE reactions

All microbiology rich media components were prepared as 10% stock solutions in 60 mM HEPES-KOH buffer pH 8, except for *P. pastoris* CFE, where distilled water was used. Four different final concentrations of these components were used in the CFE reaction, i.e., 0.4, 0.8, 1.67 and 2.5% (w/v). The list of different commercial sources and batch numbers of the component used in this research are listed in the supplementary file (Supplementary Table S1).

### Denaturing PAGE and in-gel FlAsH staining

For a 33 µL CFE sample, 1 ml of ice-cold acetone was added and incubated at -20 °C for 1 h. The sample was centrifuged at 16,000 × *g*, 10 min and the pellet was washed twice with 70% ice-cold acetone. The supernatant was removed, and the pellet was dried. To the dried pellet, 22 µL of ddH_2_O, 10 µL of 4x SDS-PAGE loading buffer, and 4 µL of 0.5 M TCEP were added and mixed by vortexing. The samples were boiled for 5 min followed by the addition of 4 µL of 1 mM FlAsH-EDT_2_ reagent. The samples were incubated at room temperature for 20 min, followed by centrifugation for 10 min, 16,000 × *g*. The supernatant was analyzed by SDS-PAGE and the gels were visualised under blue light as well as Coomasie Blue staining.

### RT-PCR

The DNA in 2 µL of CFE reaction was degraded with 0.5 µL of TURBO DNase (2 U/µL, Invitrogen, AM2238) at 37°C for 30 min. The CFE reaction was quenched by addition of 15 mM EDTA and incubated at 75°C for 15 min. The denatured proteins were pelleted through centrifugation at 3,200 x *g* for 2 minutes. To prepare a 20 µL reverse transcription reaction, 2 µL of DNase-treated sample was mixed with 1 µM reverse primer (GATCCCGGCGGC), 10 mM DTT, 0.5 mM dNTP (Denville, CB4430-2), 5 U/µl protoscript II reverse transcriptase (NEB, M0368X), 1x protoscript II reverse transcriptase buffer, and 0.4 U/µL Murine RNase Inhibitor (NEB, M0314S). The reverse transcription was performed at 42°C for 1 hour, followed by the inactivation at 65°C for 20 min. A 25 µL qPCR reaction was performed by mixing the following: 1 µL of the reverse transcribed DNA, 0.8 µM forward (AAG​TTC​ATC​TGC​ACC​ACC) and reverse (TTG​AAG​TCG​ATG​CCC​TTC) primers, 1x OneTaq Hot Start 2X Master Mix with Standard Buffer (NEB, M0484L), and 1x Chai Green Dye (CHAI, R01200S). The qPCR was performed on CFX96 Touch Real-Time PCR Detection System (BioRad). The thermocycling program was set up as follows: one cycle of 30 s denaturation at 95°C, 30 cycles of 15 s denaturation at 95°C, 15 s annealing at 54°C, 1 minute extension at 68°C, and one cycle of 5 minutes final extension at 68°C. The amplification curves plotted through CFX Maestro Software to determine Cq values and averaged across three replicates of each sample were calculated separately.

## Results

AAs are a rate-limiting factor in CFE reactions, although some residual levels are present in most cell extracts, because of the processing of cells typically at mid-exponential growth phase. The addition of AAs to CFE makes up about 8% of the overall cost (Sun et al., 2013), although this figure depends on the AA concentration (variable) and scale. Conventionally, CFE systems use commercial AAs, which are sometimes proprietary, to mitigate for solubility and pH issues. To reduce cost and simplify the reaction mixture, we considered rich media components as an alternative AA source. This is plausible since most CFE protocols require the prior growth of microbial cells on rich media, prior to cell extract harvesting. While there are free AAs present in some rich media components, microbes also secrete proteases and peptidases to catabolise these complex mixtures.

### *E. coli* CFE is active with a range of rich media sources

**FIGURE 1:**
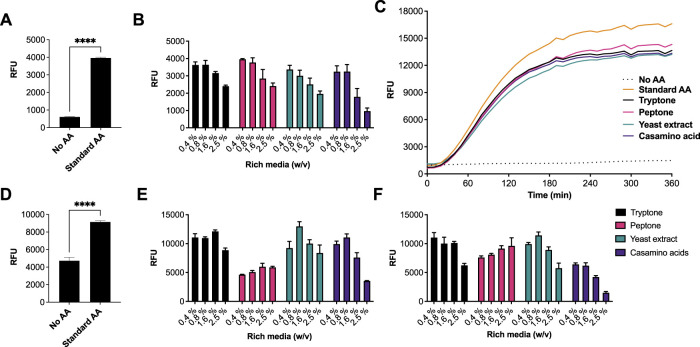
CFE activity of *E. coli* Rosetta^TM^ 2(DE3) and BL21(DE3) systems with AA substitutes. **(A)** End-point fluorescence measurements of *E. coli* Rosetta^™^ (DE3) 2 control reactions with no AAs and 1 mM of AA standard (See methods). **(B)** End-point fluorescence measurements of *E. coli* Rosetta^™^ (DE3) 2 reactions. **(C)** *E. coli* Rosetta^™^ (DE3) 2 time-course reaction. **(D)** End-point fluorescence measurements of *E. coli* BL21 (DE3) control reactions with no AAs and 1 mM of commercial standard (Sigma). **(E)** End-point fluorescence measurements of *E. coli* BL21 (DE3) reactions with rich media commercial batch A, see Supplementary Table S1. **(F)** End-point fluorescence measurements of *E. coli* BL21 (DE3) reactions with rich media commercial batch B, see Supplementary Table S1. Cell-free reactions were set-up as described in the methods and results text. Experiments were performed on two independent days to ensure reproducibility. Error bars (removed in panel C for clarity) in standard deviation represent three technical measurements. End-point samples were collected after overnight incubation (16 h) at 30°C.

We began by investigating different rich media sources to support an *E. coli* CFE system. This is because *E. coli* is the dominant CFE system used widely throughout the international cell-free synthetic biology research community and within industrial biotechnology. Therefore, we performed these experiments in both *E. coli* Rosetta^™^ 2 (DE3) (Figures 1A–C) and BL21 (DE3) strains (Figures 1D–F), since these strains are the most widely used *E. coli* CFE hosts (Sun et al., 2013; Kwon and Jewett, 2015). For the *E. coli* Rosetta^™^ 2 (DE3) CFE reactions, we used an eGFP reporter coupled to a strong T7max promoter (Sato et al., 2022). First, there was a 6.5-fold increase in relative fluorescence when comparing no AAs with the 1 mM commercial standard (*p* = 0.0001). Interestingly, all four rich media components (tryptone, peptone, yeast extract and casamino acids), at a concentration of 0.4–0.8% (w/v), produced equivalent amounts of eGFP compared to the 1 mM commercial AA mixture. In contrast, concentrations above 1.67% (w/v) decreased eGFP production by approximately 40–60% (Figure 1A). In addition, we monitored fluorescent protein production over a time course. This determined that the rate of protein synthesis across assays carried out with different amino acid sources was broadly unchanged (Figure 1C). To verify whether there were any changes in mRNA abundance in the *E. coli* Rosetta^™^ 2 (DE3) CFE experiments, quantitative RT-PCR was performed. This experiment confirmed that none of the alternative AA sources altered mRNA expression (Supplementary Figure S1). We also repeated these experiments in a *E. coli* BL21 (DE3) CFE system using a mScarlet-I reporter coupled to a strong constitutive synthetic promoter 44 (SP44) (Bai et al., 2015), which is highly active in both *E. coli* and *Streptomyces* CFE. For this, we observed broadly similar findings between the two different approaches and expression constructs (Figure 1C). Protein yields of CFE reactions with 0.4–0.8% (w/v) of alternative AA source were equivalent to that of CFE with 1 mM conventional AA mix. However, while there was significant variability between different companies/batches (Figures 1E,F), at least one batch (codes listed in supplementary information), gave equivalent activity to the commercial AA source for both the Rosetta^™^ 2 (DE3) and BL21 (DE3) CFE systems.

### Tryptone and casamino acids provide strong *Streptomyces* CFE activity

**FIGURE 2:**
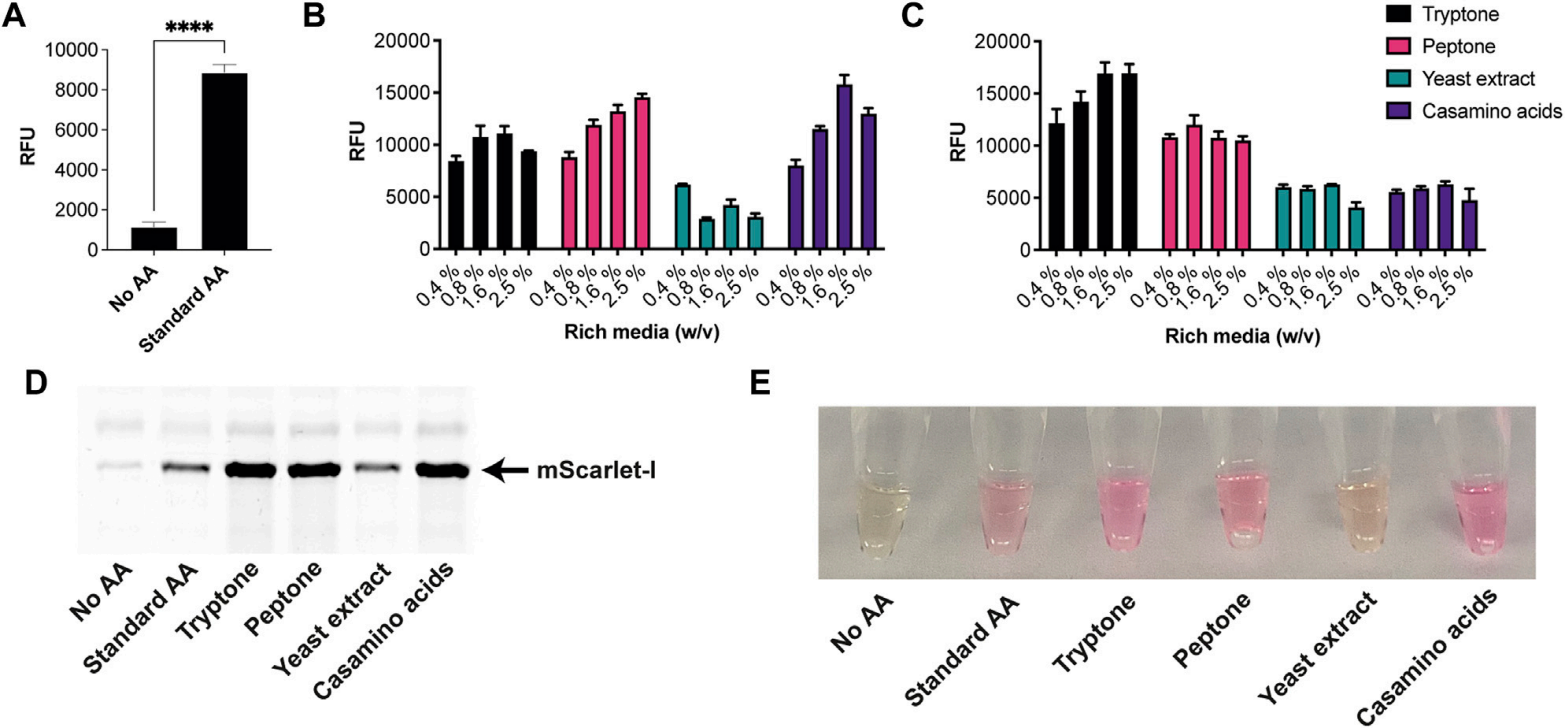
*S. venezuelae* CFE activity with AA substitutes. **(A)** End-point fluorescence measurements of *S. venezuelae* CFE control reactions with no AAs and 1 mM of commercial standard (Sigma). **(B)** End-point fluorescence measurements of *S. venezuelae* CFE reactions with rich media commercial batch A, see Supplementary Table S1. **(C)** End-point fluorescence measurements of *S. venezuelae* TX-TL reactions with rich media commercial batch B, see Supplementary Table S1. **(D)** In-gel fluorescence stain of C-terminal tetracysteine tagged mScarlet-I. **(E)** Visual image of mScarlet-I at endpoint. Cell-free reactions were set up as described in the methods and results text. Error bars in standard deviation represent three technical measurements. End-point samples were collected after overnight incubation (16 h) at 30°C.

**FIGURE 3:**
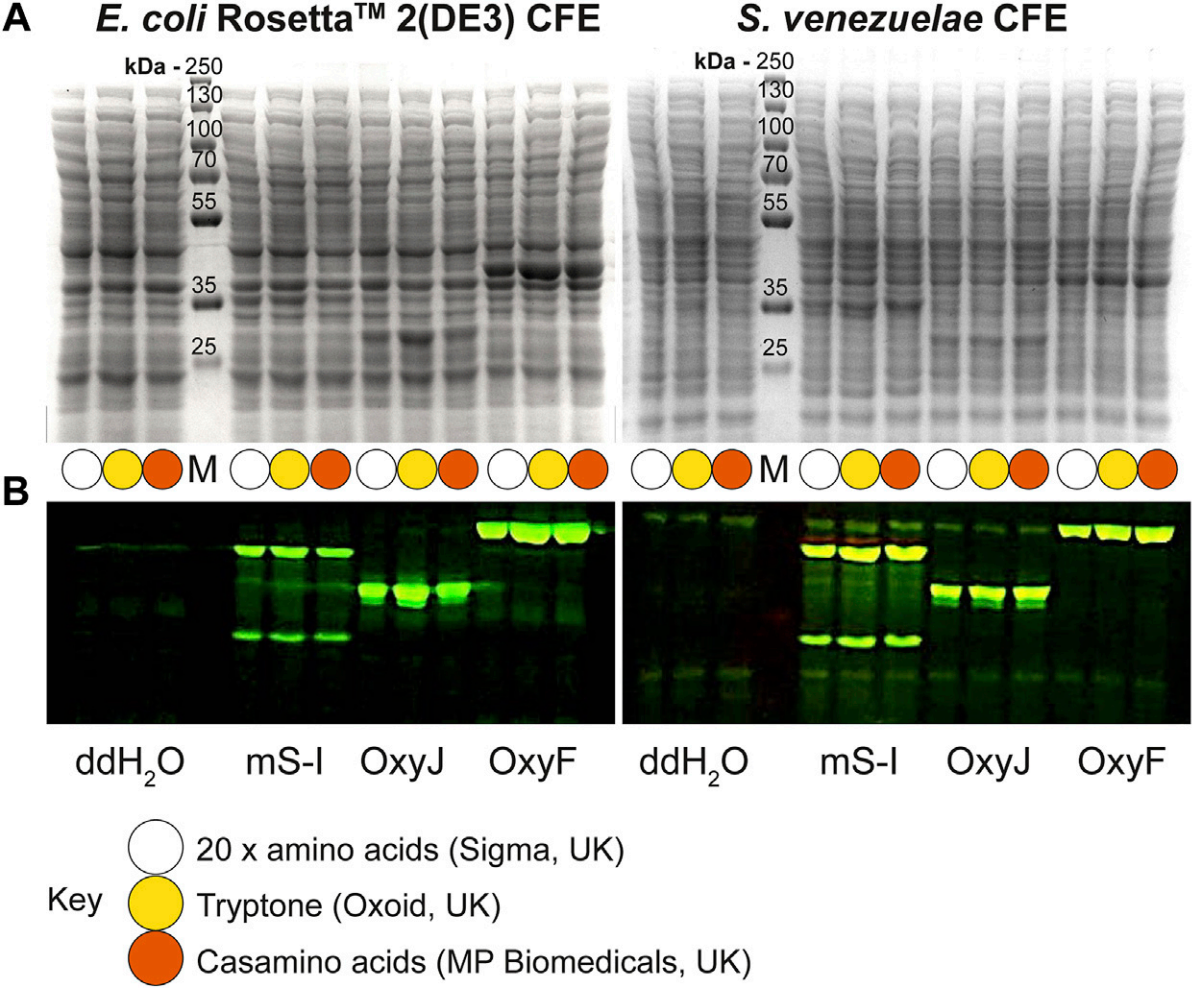
Denaturing PAGE analysis of *E. coli* Rosetta^™^ 2(DE3) and *S. venezuelae* CFE reactions making mScarlet-I, OxyJ and OxyF proteins using different AA sources. **(A)** Coomassie blue staining and **(B)** In-gel fluorescence stain of C-terminal tetracysteine tagged proteins. The best-performing batches for tryptone (Batch B) and casamino acids (Batch A) at 1.6% (w/v) were used to compare with the standard AAs source. Abbreviations: Pre-stained PageRuler™ (Thermo Scientific) protein marker (M); mScarlet-I (mS-I).

Next, we tested the *S. venezuelae* CFE system, which we have specifically optimised to study high G + C genes from actinomycetes genomes. Here, we also used the SP44-*mScarlet-I* expression plasmid, optimised for *S. venezuelae* CFE (Bai et al., 2015). Remarkably, we observed strong production of mScarlet-I with tryptone, at up to 17 μM (0.45 mg/ml). This simple protocol modification provided up to 2-fold increase in activity (*p* = 0.0002) compared to the commercial AA source (Figures 2A–C), which was also observed through in-gel fluorescence staining of the C-terminal tetracysteine tagged mScarlet-I (Figure 2D) and visually (Figure 2E). However, an equivalent batch of tryptone from a different commercial source did not provide a significant increase in CFE activity (Figure 2B). Therefore, we repeated these experiments across several rich media sources, such as peptone, yeast extract, and casamino acids. In summary, while there is variation between commercial batches, tryptone and casamino acid consistently produced the highest yields of mScarlet-I protein yield. For comparison, the commercial AA source produced 8 µM mScarlet-I, while peptone yielded 14 µM mScarlet-I (Figure 2B). Yeast extract was the least effective AA source for *Streptomyces* CFE. It was surprising to observe CFE activity for casamino acids, since free L-tryptophan (L-Trp) is depleted during the manufacturing process. To test this, we compared *Streptomyces* CFE reactions with standard AA, to reactions lacking L-Trp. This only reduced the CFE activity by 48%, suggesting significant levels of L-Trp are present in the cell extract. Alternatively, if 1 mM L-Trp was supplemented to CFE reactions run with casamino acids, there was no significant change in activity. This suggests L-Trp is not sufficiently limiting in the reaction. In addition, we performed high-performance liquid chromatography analysis on two batches of casamino acids in comparison with a L-Trp standard. Casamino acids batch A (MP Biomedicals, United Kingdom), the best performing batch for CFE activity (Figure 2B), contained approximately 1 mM of L-Trp in 2% (w/v). L-Trp was not detected in batch B (Oxoid, United Kingdom), although CFE activity was equivalent to standard amino acids. In addition, to verify whether our findings were compatible with other model proteins, we compared the relative CFE production of two *Streptomyces* enzymes, OxyJ and OxyF (Moore et al., 2017a). Here, we tested the best performing casamino acids and tryptone batches with both *Streptomyces* and *E. coli* Rosetta^™^ (DE3) 2 CFE. In-gel fluorescence and Coomassie blue staining, showed strong production of both proteins, at least equivalent to levels observed with standard AA in both *E. coli* Rosetta^™^ (DE3) 2 and *Streptomyces* CFE systems (Figure 3). This data overall suggests casamino acids and tryptone are an effective AA replacement for recombinant protein production purposes.

### Rich media is active in *P. pastoris* CFE

**FIGURE 4:**
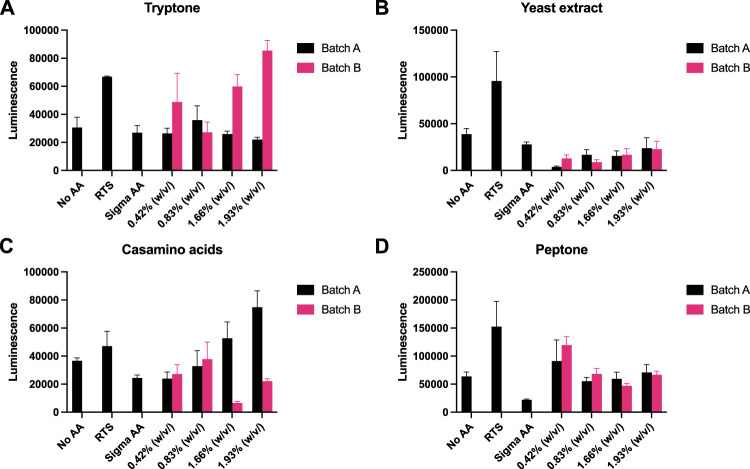
*P. pastoris* CFE with AA substitutes. **(A)** End-point luminescence measurements of *P. pastoris* CFE with **(A)** tryptone, **(B)** yeast extract, **(C)** casamino acids and **(D)** peptone. Error bars represent standard deviation of three technical measurements. End-point samples were collected after 2 h of incubation at 30°C.

Recently, eukaryotic CFE models have been optimised to provide a strong homologous system to make challenging eukaryotic proteins (Spice et al., 2020; Zhang et al., 2020) and for the study of cell-free prototyping (Kopniczky et al., 2020). To extend our findings, we repeated our experiments with *P. pastoris*, an emerging eukaryotic CFE expression system (Thoring et al., 2016; Zhang et al., 2020). For this system, we followed previous published literature, and used a luciferase reporter for detection of protein synthesis (Spice et al., 2020; Spice et al., 2022). Due to the stability of luminescence assays, the controls (no AA, RTS and Sigma AA) were tested separately against each rich media component. Across the four rich media options (tryptone, yeast extract, casamino acids and peptone), we observed broadly similar findings to the prokaryotic CFE results. Interestingly, while luminescence was observed in the absence of added AA, in comparison, the RTS AA standard, tryptone or casamino acids, showed increased activity (two to four-fold). Like the *E. coli* and *Streptomyces* CFE systems, this effect was also batch dependent(Figure 4).

## Discussion

We have previously tested a variety of AA sources and methods in different prokaryotic CFE systems, with variable findings. While *E. coli* CFE systems are robust, we found that the activity of alternative CFE systems (e.g., *Streptomyces*/*Bacillus*) were sensitive to the salts or proprietary reagents required for AA stock preparation (Moore et al., 2018; Moore et al., 2021). Therefore, we desired a simple AA source for general use in CFE systems. Rich media are ubiquitous AA sources used for general microbial growth, and contain free AAs, short peptides, proteolytic fragments, and, depending on the source (e.g., yeast extract) also vitamins, cofactors, and primary metabolites. In summary, specific batches (Supplementary Table S1) of tryptone and casamino acids gave up to a 2-fold increase in protein yield (up to 0.45 mg/ml) for the *S. venezuelae* CFE system (Moore et al., 2021), which is of specific interest for studying high G + C (%) genes from related microbial genomes. In the *E. coli* and *P. pastoris* CFE systems we tested, while there was no general preference for AA source, there was at least equivalent activity for when the commercial AA standard mixture was replaced with rich media. Our experiments also show that each CFE system has specific preferences for rich media source, while there is also variation in activity between commercial brands and batches. Batch variation is an important limitation of our study, likely due to different AA frequencies. However, *E. coli* CFE reactions were shown to be more productive with a fixed AA concentration, in comparison to a mixture adjusted close to the distribution of AAs in *E. coli* cells (Lobry and Gautier, 1994; Caschera and Noireaux, 2015b). Furthermore, our finding was reproducible in three separate laboratories (United Kingdom and USA) with different CFE systems and commercial rich media sources. Considering batch variation, we suggest that while rich media may not be suitable for all cell-free experiments, especially where modelling or metabolomics is important, our method provides a cost-effective AA source with potential application for high-value recombinant protein production. For example, a typical 0.5 kg unit of rich media will provide a long-lasting and low-cost AA source for CFE–approximately 1–2 g rich media (∼$6) is required per litre of CFE reagent. Therefore, this method advancement has a clear cost benefit over commercial AA sources. Compared to the widely used RTS AA kit (Biotech Rabbit), our method provides up to a 300-fold reduction in cost for the AA source (Supplementary Table S2). Depending on scale and format, AAs cost approximately 8% of the overall CFE reaction. Finally, rich media components are available in most biological laboratories. Therefore, our method is easily accessible and provides an improvement that lowers the threshold for engagement with cell-free systems, which require multiple components and where access to some laboratory reagents is limiting for some research groups. Therefore, we believe our method advancement will widen interest in cell-free systems in both academic and educational settings (Huang et al., 2018), as well as provide a cost-benefit to those interested in using CFE systems for high-value recombinant protein production.

## Data Availability

The original contributions presented in the study are included in the article/Supplementary Material, further inquiries can be directed to the corresponding author.
