# Supplementary material for: A ubiquitous amino acid source for prokaryotic and eukaryotic cell-free transcription-translation systems
Source: Front Bioeng Biotechnol. 2022 Sep 16;10:992708. doi: 10.3389/fbioe.2022.992708 (PMC9524191; doi:10.3389/fbioe.2022.992708)
Supplement: Supplementary file 1 [file DataSheet1.pdf]

Supplementary information

A ubiquitous amino acid source for prokaryotic and eukaryotic cell-free transcription-translation systems

Lakshmeesha K. Nagappa[1], Wakana Sato[2], Farzana Alam[3], Kameshwari Chengan[1], Christopher M. Smales[1], Tobias von der Haar[1], Karen M. Polizzi[3,4], Katarzyna P. Adamala[2], and Simon J. Moore[1*]

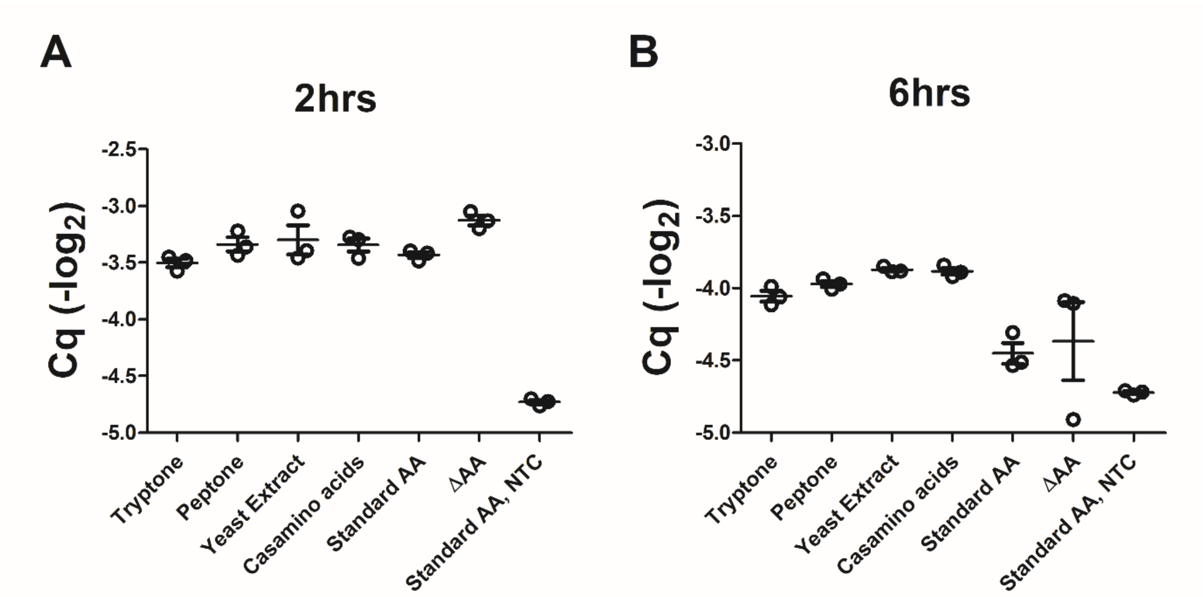

Figure S1: mRNA abundance in *E. coli* Rosetta2 CFE. qPCR measurement of CFE after A) 2 and B) 6 hours incubation. NTC is a negative control without eGFP plasmid. ΔAA is the reaction without amino acid sources. NTC is the 'no DNA control'. Cq - the quantification cycle. The graph shows means with error bars that signify SEM (n = 3).

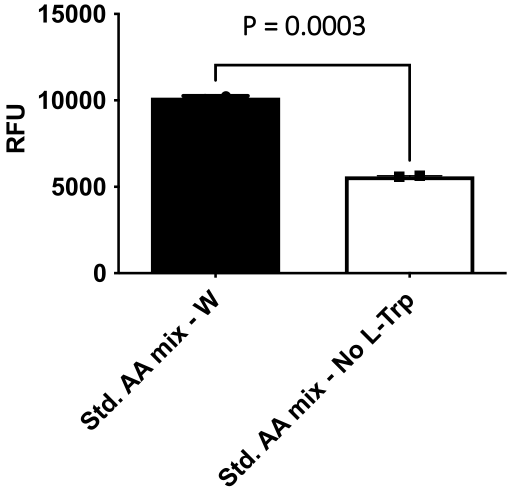

Figure S2: *S. venezuelae* CFE activity with and without 1 mM L-Trp in a reaction containing 19 x standard (Std.) amino acids (AA). Average and standard deviation are representative of two independent measurements.

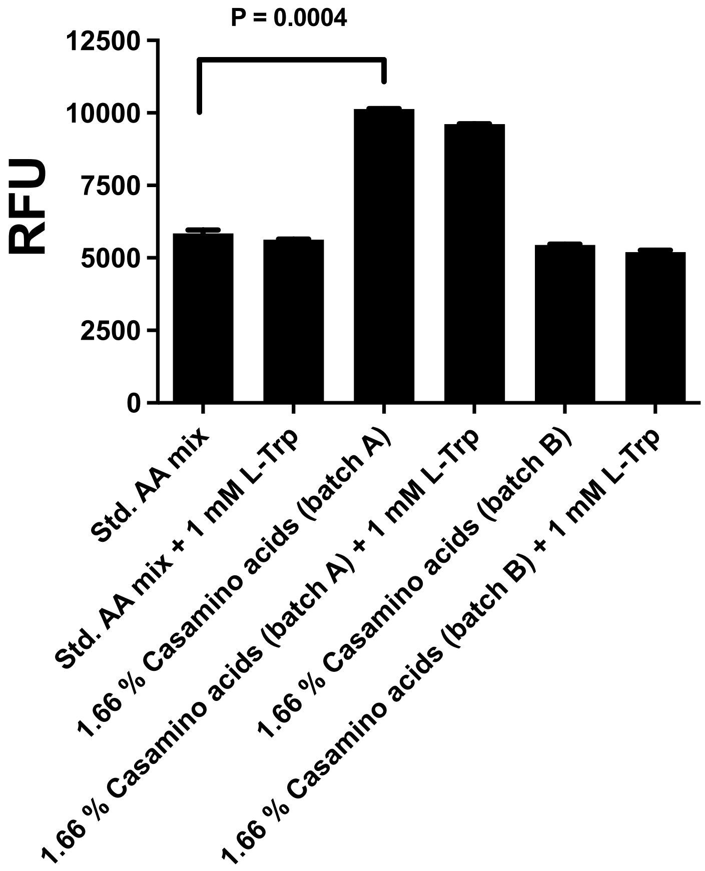

Figure S3: Figure S2: *S. venezuelae* CFE activity with and without 1 mM L-Trp supplemented with different amino acids sources. Average and standard deviation are representative of three technical replicates.

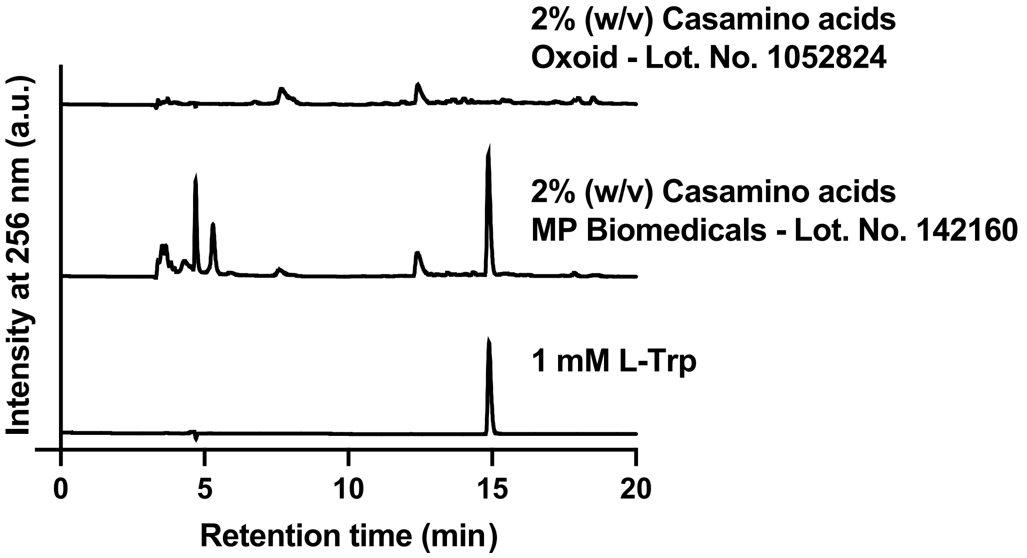

Figure S4. High-performance liquid chromatography analysis of L-Trp standard and casamino acid batches. Separation was performed Agilent 1100 liquid chromatography system using an ACE 5 AQ C-18 column (2.1 x 250 mm, 5 mm particle size). For HPLC analysis, buffer A was

water with 0.1% (v/v) trifluoroacetic acid and buffer B was acetonitrile, for a total run time of 60 min including 10 min post-run time. 10mL samples were injected, and the column was washed at 0.2 mL/min with the following gradient: 0 min 5% B, 5 min 20% B, 50 min 70% B, 52 min 100% B, 58 min 100% B and 60 min 5% B. Ultraviolet-visible (UV-Vis) light spectra and set wavelengths (256 and 280 nm) were recorded by a diode array detector.

Table S1: List of different batches/brands of amino acids sources used in Figure 1-3.

**Batch**	**Reagent**	**Form**	**Brand**	**Lot. No**	**Cat No.**
**A**	Tryptone	Granulated	Melford	50297-51481	T60065-2000.0
**B**	Tryptone	Powder	Oxoid	246147	LP0042
**A**	Peptone	Powder	BD	8218523	211677
**B**	Peptone	Powder	Oxoid	664733	LP0037
**A**	Yeast extract	Granulated	Melford	49133-50836	Y20025-2000.0
**B**	Yeast extract	Powder	Melford	52892-54435	Y20020-500.0
**A**	Casamino acids	Powder	MP biomedicals	142160	3060012
**B**	Casamino acids	Powder	Oxoid	1052824	LP0041

Table S2: Cost of different amino acid sources used in CFE reactions.

**Name of the reagent**	**RTS amino acid**	**Tryptone**	**Casamino acids**
Source	Biotechrabbit	Oxoid	MP biomedicals
Cat. No.	BR1401801	LP0042B	3060012
Quantity	1.5 mL (168 mM) *20 amino acids	500 grams	227 grams
Cost per unit*	$378	$175	$75
Cost/mL TX-TL	$1.52	$0.006	$0.005
Fold reduction in cost compared to RTS kit	-	253	304

*Cost is dollars is calculated as per the exchange rate and sales prices on 2nd May 2022.

Supplementary methods

Preparation of *E. coli* Rosetta / BL21 2 cells - adapted from Sun *et al* (1)

A 50 mL starter culture of the Rosetta 2 / BL21 strains of *E. coli* (Millipore Sigma 71400-3) was made from a glycerol stock and grown at 37˚C overnight in 2xYPTG (16 g/L tryptone, 10 g/L yeast extract, 5 g/L NaCl, 20 mM glucose, 40 mM potassium phosphate dibasic, 22 mM potassium phosphate monobasic) with 34 mg/mL chloramphenicol.

A 5 mL of the starter culture was inoculated in a 750 mL 2xYPTG (without chloramphenicol). The culture was grown at 30˚C to an OD_{600} of 0.4-0.6, then harvested. The resulting pellet was washed twice with 200 mL of Wash Buffer (10 mM Tris acetate pH 8.2, 14 mM magnesium acetate, 60 mM potassium acetate, 2 mM DTT) followed by a third wash of 40 mL. The resulting cell pellet was flash frozen in liquid nitrogen and stored at -80˚C prior to the following steps.

Cell lysis - adapted from Kwon *et al* (2)

Frozen pellets were suspended in cold Wash Buffer equivalent to 1.1 times the cell mass. The cells were then lysed by sonication at 4˚C with total output energy of 1.7 kJ per 4.5 mL suspension, using the following sonicator settings: 50% amplitude, repeating 10 s on followed by 15 s off. The lysate was centrifuged at 15,000 × *g* at 4˚C for 30 min. The supernatant was split into 500 mL fractions and incubated at 37˚C with shaking for 1 h. The lysate was then centrifuged at 15,000 × *g* at 4˚C for 30 min. The lysate was prepared as single-use aliquots, flash frozen, and stored at -80˚C until further use. A Bradford assay was also performed to determine protein concentration (~typically 28-32 mg/mL), as described (3).

Preparation of the *E. coli* Rosetta 2 reaction - adapted from Sun *et al* (1)

The reaction is composed of the following: 12 mM magnesium glutamate, 140 mM potassium glutamate, 1 mM DTT, 1.5 µM T7 RNA polymerase (prepared in house), 0.4 U/µl Murine RNase Inhibitor (NEB, M0314S), 10 mg/mL *E. coli* cell-extract, 1 x energy mix, and different amino acid sources. The eGFP plasmid concentration was 10 nM. The reaction was incubated at 30˚C for 6 hours followed by 4˚C hold for endpoint eGFP measurement. For qPCR experiment, the TX-TL reaction was incubated at 30˚C for 2 or 6 hours.

The 10 x energy mix is composed of the following: 500 mM HEPES-KOH pH 8, 15 mM ATP, 15 mM GTP, 9 mM CTP, 9 mM UTP, 2 mg/mL *E. coli* tRNA, 0.68 mM folinic acid, 3.3 mM NAD, 2.6 mM CoA, 15 mM spermidine, 40 mM sodium oxalate, 7.5 mM cAMP, 300 mM 3-PGA. The 20 mM standard amino acid stock is composed of 20 mM of the following amino acids: alanine, arginine, asparagine, aspartic acid, cysteine, glutamic acid, glutamine, glycine, histidine, isoleucine, leucine, lysine, methionine, phenylalanine, proline, serine, threonine, tryptophan, tyrosine, and valine. Those amino acids were dissolved in pH 6.5, 400 mM potassium hydroxide solution. The alternative amino acid sources were dissolved in 60 mM HEPES-KOH pH 8.0. Chemicals were sourced as previously listed (1), or as stated in the main methods text.

Preparation of the *E. coli* BL21 2 reaction - adapted from Sun *et al* (1)

The reaction was the same as the Rosetta 2 reaction described above, with the exception that T7 RNA polymerase and murine RNase inhibitor were not included. The mScarlet-I plasmid concentration was set at 10 nM and was measured as an end-point sample after 16 hours incubation at 30˚C.

***S. venezuelae* CFE protocol.**

For detailed protocol guidance see Toh *et al* (3). A single colony of *S. venezuelae* was inoculated into 5 mL of GYM (4 g/L D-glucose, 4 g/L yeast extract and 10 g/L malt extract at pH 7.5 with KOH base) and incubated overnight at 30˚C, 200 rpm. 1 mL of the overnight culture was inoculated into 50 mL of fresh GYM and incubated at 30˚C, 200 rpm till A_{600} of 3-4 was reached. 0.25 mL of this culture was inoculated into 1 L of GYM in a 2 L baffled flask and incubated at 30˚C, 200 rpm for 14-16 hours. Cells were harvested at A_{600} of 3-4 by centrifugation. The cells were washed using cold S30A (10 mM HEPES KOH pH 7.5, 10 mM $MgCl_2$, 1 M NH_4Cl, and 2 mM DTT) and S30B (50 mM HEPES KOH pH 7.5, 10 mM $MgCl_2$, 50 mM NH_4Cl, and 2 mM DTT) buffers sequentially. The resulting pellet was resuspended in 0.9 volumes of S30B buffer and sonicated at 4˚C using the following parameters: 65 % amplitude, 10s on, 10s off and an energy input of 240 kJ per mL of cell suspension. The lysate was centrifuged at 16,000 × *g* for 10 min at 4 °C to remove the cell debris. The supernatant was subjected to a run-off reaction at 30˚C for 60 min followed by centrifuged at 16,000 × *g* for 10 min at 4 °C. The supernatant was split into 1 mL aliquots, flash frozen and stored at -80˚C. A Bradford assay was also performed to determine protein concentration (~typically >22-24 mg/mL), as described (3).

The *S. venezuelae* CFE reaction is composed of the following: 25 mM HEPES-KOH pH 8, 8.33 mM magnesium glutamate; 150 mM potassium glutamate, 1% (w/v) polyethylene glycol 6000, 1 mM nucleotide monophosphates (AMP, UMP, GMP, CMP), 30 mM 3-PGA, 5 mM glucose-6-phosphate, 1.5 mg/mL polyvinyl sulfonic acid, 1 x cell-free extract, and different amino acid sources – standard amino acid mix 1 mM or different percentages of alternate amino acid sources whose stock was 10 % w/v made in 60 mM HEPES-KOH pH 8. 1 M stocks of 20 standard amino acids were made by dissolving each amino acid in their compatible solvent as mentioned by the manufacturer (Sigma Aldrich - Product. No. 09416). A 6 mM amino acid mixture was made in 100 mM KOH. The mScarlet-I plasmid concentration was 20 nM. The reaction was incubated at 30˚C for 16 hours followed by end-point measurement.

***P. pastoris* CFE protocol**

The cell-free extract preparation, reaction and luminescence measurements is identical to recent literature (4–6).

1. Z. Z. Sun, *et al.*, Protocols for implementing an Escherichia coli based TX-TL cell-free expression system for synthetic biology. *J. Vis. Exp.* **50762**, e50762 (2013).

2. Y.-C. Kwon, M. C. Jewett, High-throughput preparation methods of crude extract for robust cell-free protein synthesis. *Sci. Rep.* **5**, 8663 (2015).

3. M. Toh, K. Chengan, T. Hanson, P. S. Freemont, S. J. Moore, A High-Yield Streptomyces Transcription-Translation Toolkit for Synthetic Biology and Natural Product Applications. *J. Vis. Exp.* (2021) https:/doi.org/10.3791/63012.

4. R. Aw, A. J. Spice, K. M. Polizzi, Methods for expression of recombinant proteins using a Pichia pastoris cell-free system. *Curr. Protoc. Protein Sci.* **102**, e115 (2020).

5. A. J. Spice, R. Aw, K. M. Polizzi, Cell-Free Protein Synthesis Using Pichia pastoris. *Methods Mol. Biol.* **2433**, 75–88 (2022).

6. A. J. Spice, R. Aw, D. G. Bracewell, K. M. Polizzi, Improving the reaction mix of a Pichia pastoris cell-free system using a design of experiments approach to minimise experimental effort. *Synth. Syst. Biotechnol.* **5**, 137–144 (2020).
